# Interaction of RARRES1 with ICAM1 modulates macrophages to suppress the progression of kidney renal clear cell carcinoma

**DOI:** 10.3389/fimmu.2022.982045

**Published:** 2022-10-24

**Authors:** Xiaodong Geng, Kun Chi, Chao Liu, Zhangning Fu, Xu Wang, Liangliang Meng, Hanfeng Wang, Guangyan Cai, Xiangmei Chen, Quan Hong

**Affiliations:** ^1^ Department of Nephrology, First Medical Center of Chinese People's Liberation Army (PLA) General Hospital, Nephrology Institute of the Chinese People’s Liberation Army, State Key Laboratory of Kidney Diseases, National Clinical Research Center for Kidney Diseases, Beijing, China; ^2^ Beijing Key Laboratory of Kidney Disease Research, Beijing, China; ^3^ Beidaihe Rehabilitation and Recuperation Center, Chinese People’s Liberation Army Joint Logistics Support Force, Qinhuangdao, China; ^4^ Department of Radiology, First Medical Centre of Chinese People’s Liberation Army (PLA) General Hospital, Beijing, China; ^5^ Department of Urology, Third Medical Center of Chinese People’s Liberation Army (PLA) General Hospital, Beijing, China

**Keywords:** kidney renal clear cell carcinoma, RARRES1, ICAM1, bioinformatic analyses, M1 macrophages

## Abstract

**Background:**

RARRES1 is a tumor suppressor protein, and its expression is suppressed in various tumor cells. However, whether it participates in the immune response in kidney renal clear cell carcinoma (KIRC) is unknown, and the defined mechanism is not clear. Therefore, the mechanism of RARRES1 in KIRC is worthy of investigation.

**Methods:**

We analysed the expression and function of RARRES1 with The Cancer Genome Atlas (TCGA) database. The Kaplan–Meier curve was adopted to estimate survival. RARRES1-correlated genes were obtained from the UALCAN database and subjected to Gene Ontology (GO) enrichment and protein–protein interaction (PPI) network analyses. The correlation analysis between tumor-infiltrating immune cells and selected genes were performed with TIMER database. We also investigated the possible function of RARRES1 in KIRC by coculturing Caki-1 cells with THP-1 cells. Immunofluorescence assay was performed to study the RARRES1 expression in difference grade KIRC tissues.

**Results:**

The expression of RARRES1 was negatively correlated with survival in KIRC patients. The GO biological process term most significantly enriched with the RARRES1-correlated genes was regulation of cell adhesion. ICAM1, which exhibited a relatively highest correlation with RARRES1, is positively correlated with the infiltration level of macrophages. RARRES1 could enhance the expression of ICAM1 in Caki-1 cells and then induce the activation of M1 THP-1 cells to decrease the viability and induce the apoptosis of Caki-1 cells.

**Conclusion:**

RARRES1 plays an antitumor role by promoting ICAM1 expression and inducing the activation of M1 macrophages. We offer insights into the molecular mechanism of KIRC and reveal a potential therapeutic target.

## Introduction

The most common form of renal cancer is known as renal cell carcinoma (RCC). It represents over 90 percent of all renal cancer cases. RCC is an immunogenic tumor characterized by frequent invasion of tumor tissues by immune cells, rare spontaneous regression, and a clinical response to immunotherapy (1). The most aggressive RCC in adults is kidney renal clear cell carcinoma (KIRC) (2). Genetic aberrations and the tumor environment have been indicated to be associated with KIRC. Extensive studies have examined the mechanisms of relapse and metastasis, but the cause and pathogenesis of KIRC is still unknown. It is worth noting that the occurrence and development of KIRC reveal a correlation with its immune microenvironment (3). The characteristic of tumor microenvironment strongly influence disease biology and may influence the response rate to systemic therapy. Targeted immunotherapy is currently an option in first line treatment because KIRC is also considered an immunogenic tumour with high numbers of immune cells (4). Macrophage infiltration in KIRC is associated with prognosis (5). Macrophages are the regulators of tumor immunity and immunotherapy (6). Macrophages can be affected by different factors to polarize toward the M1 or M2 phenotype and thus affect tumor progression (7). It is necessary to unravel the causes and mechanisms to find biomarkers for diagnosis or personalized therapy of KIRC (8).

RARRES1 was described as a retinoid response gene in skin raft cultures and thought to be a transmembrane protein (9). RARRES1 is induced by retinoic acid and expressed in multiple tissues. Research has shown that RARRES1 inhibits tumor cell proliferation or invasion and induces apoptosis of tumor cells (10). RARRES1 has been shown to act as an invasion inhibitor in prostatic tumor cell lines (11). In inflammatory breast cancer, RARRES1 mediates the regulatory cancer invasion cells through the Axl pathway (12). RARRES1 has been described as a cell adhesion molecule that increases cell contact or reduces cell proliferation. Previously, downregulation of RARRES1 has been confirmed in some human cancers (13). RARRES1 is epigenetically silenced by promoter methylation, and its promoter is hypermethylated in prostate, nasopharyngeal, and gastric cancers (14). Hence, understanding the regulatory mechanism and molecular functions of RARRES1 may identify potential targets for the diagnosis and immunotherapy of KIRC.

## Materials and methods

### Clinical cohorts and RNA-Seq datasets

RNA-seq datasets, including information on the clinical cohorts, were searched from TCGA database (http://cancergenome.nih.gov/). 533 patients of KIRC and 72 controls were enrolled in this study. The clinical features of these data included patient gender, race, age and survival time.

### Analysis of RNA-Seq data

Differential expression analysis and survival analysis of Kaplan–Meier(KM) curve were performed to compare the normal control group and KIRC patients using the UALCAN tool (http://ualcan.path.uab.edu/) (15). The bioinformatic analyses of RARRES1-correlated genes included Gene Ontology (GO) enrichment and protein–protein interaction (PPI) analyses with the Metascape analysis tool (http://metascape.org/) (16). All of these analytical tools can be accessed online.

### Immune infiltration analysis

Tumor Immune Estimation Resource (TIMER; http://cistrome.shinyapps.io/timer/) (17) was adopted to carry out a comprehensive correlation analysis of RARRES1 expression with the features of tumor-infiltrating immunocytes (TILs) in KIRC. The “Gene module” of TIMER could visualize the correlation of gene expression with immune infiltration level in KIRC. The scatterplots will show the purity-corrected partial Superman’s rho value (partial.cor) and statistical significance(P value). The integrated repository portal for tumor-immune system interactions (TISIDB; http://cis.hku.hk/TISIDB) (18) was used to inspect tumor-immune system interactions for 28 TILs in human cancers. For each cancer type, the relative abundance of TILs were inferred by using gene set variation analysis (GSVA) based on gene expression profile. Spearman correlation analysis was used to assess the relations between RARRES1 and TILs in TISIDB. The correlations between RARRES1 expression and hub gene expression in KIRC were determined using Gene Expression Profiling Interactive Analysis (GEPIA; http://gepia.cancer-pku.cn) and was calculated by Pearson correlation coefficient.

### Cell culture and Transwell assay

Both the human KIRC cell line Caki-1 and the human macrophage line THP-1 were purchased from American Type Culture Collection(ATCC company). THP-1 cells and Caki-1 cells were all cultured in Dulbecco’s modified Eagle’s medium (DMEM) (HyClone™) supplemented with 10% fetal bovine serum (FBS) (Gibco™) at 37°C with 5% CO_2_ in a humidified incubator. To assess the interaction between macrophages and RCC cells, Caki-1 cells and THP-1 cells were co-cultured in a Transwell system (0.4 μm pore diamete, Corning Incorporation) in a 6-well plate for 24 h, allowing free diffusion of molecules between the two compartments but not cell translocation. THP-1 cells were seeded in the upper chamber of Transwell system and co-cultured with Caki-1 cells in the bottom chamber.

### Induction and identification of macrophages

THP-1 cells were treated with 100 ng/mL PMA (#HY-18739, MCE) for 24 h every 2 days to induce differentiation into M0 macrophages. To induce M1 macrophage polarization, M0 macrophages were exposed to 20 ng/mL IFN-γ(#HY-p7025, MCE) and 100 ng/mL LPS (#HY-D1056, MCE) for 24 h. Cells were collected for qRT-PCR detection and western blot analysis. The markers of M0 macrophage (CD68) and M1 macrophage(CD86) were analyzed by western blot in THP-1 cell. And the markers of M1 macrophage(CD86) was also analyzed by qPCR method.

### Lentivirus construction and cell infection

The full-length RARRES1 cDNA fragment was cloned into the lentiviral vector pReceiver-lv185 by Guangzhou FulenGen Co., Ltd; empty vector was regarded as negative control. The pReceiver-RARRES1 lentivirus was produced by 293T cells. Before cell transduction, Caki-1 cells digested into single cell suspension were inoculated into 6-well culture plates and cultured in cell culture incubator. Then, Caki-1 cells were infected with pReceiver-RARRES1 lentivirus(RARRES1-OE group) and empty vector(Vector group) when 70% to 80% confluent respectively; Caki-1 cells without any intervention served as a blank control(control group). Quantitative PCR was used to evaluate the mRNA expression level of RARRES1 and RARRES1 related-ICAM1 in Caki-1 cells.

### RNA extraction and quantitative PCR

THP-1 cells (macrophages) or Caki-1 cells (RCC) were grown in 6-well plates. THP-1 cells were cultured for 24 h after being transfected with either control or siRNA-CD11b. Caki-1 cells transfected with control or siRNA-ICAM1 were cultured for another 24 h. TRIzol reagent purchased from Invitrogen Life Technologiesis was used to isolate total RNA from cells. A first-strand reverse transcription kit (Thermo Fisher, Carlsbad, CA) was used to synthesize complementary DNA, and SYBR Green dye mixture (Takara, Kusatsu, JPN) was used for qPCR with the following primers:

RARRES1, 5’-TGGCTTTCCTTGGAAGCTCT-3’(Forward)
and 5’-AGGTTTTTCTTACCCACTGCCT-3’(Reverse);
ICAM1, 5’-ACGGAGCTCCCAGTCCTAAT-3’ (Forward)
and 5’-CTCCTTCTGGGGAAAGGCAG-3’ (Reverse);
CD86, 5’-AGCTTTGCTTCTCTGCTGCTGTA-3’ (Forward)
and 5’-CAGCACCACTGGGGATCCATTT-3’ (Reverse);
CD11b, 5’-CCCAATTGTGACCGCAAAGG-3’(Forward)
and 5’-GGCAGCTTCATCCCGTACTT-3’(Reverse).

The siRNAs targeting human CD11b and ICAM1 and the NC siRNA (Si-CTRL) were purchased from RiboBio company. The oligonucleotide sequences were as follows:

si-CTRL (Nontargeting), 5’-UUCUCCGAACGUGUCACGUTT-3’ (Forward) and 5’-ACGUGACACGUUCGGAGAATT-3GUGACACGUU (Reverse);
siRNA-CD11b, 5’-AUCAAGAAGGCAAUGUCACUA-3’ (Forward) and 5’-GUGACAUUGCCUUCUUGAUUG-3’ (Reverse);
siRNA-ICAM1, 5’-UUGAAUAGCACAUUGGUUGGC-3’ (Forward) and 5’-CAACCAAUGUGCUAUUCAAAC-3’ (Reverse)

### ELISA detection of ICAM1

Twenty-four hours after M1 THP-1 cells and Caki-1 cells were cocultured in the Transwell system, the concentration of ICAM1 in the cell culture supernatant was determined by ELISA following the kit instructions (#ab100688, Abcam).

### Co-Immunoprecipitation

THP-1 cells treated in three different ways (THP-1 cells only, THP-1 cells cocultured with Caki-1 cells in transwell system after 24h and THP-1 cells cocultured with RARRES1-overexpression (OE) Caki-1 cells in transwell system after 24h were collected separately. Co-IP assays were carried out using the Pierce CoImmunoprecipitation(Co-IP) Kit (#26149,Thermo Scientific™,MA, USA) according to the manufacture’s instruction. The antibodies used were as follows: IP: (Mac-1) CD11b/Integrin αM Polyclonal antibody(#21851-1-AP; Proteintech). The Mac-1 protein expression of THP-1 cells in three groups were tested by western blot analysis. IgG group was used as a negative control. Extracted cell lysates before adding antibodies (imput) were also used as a control for target protein-GAPDH detection.

### Immunofluorescent staining

The studies involving human participants were approved by the PLA General Hospital ethics committee (Approval Number S2015-061-01). The patients had provided informed consent when their tissues were stored in the tissue bank. The KIRC tissues which fixed in formalin and made into paraffin embedded tissue blocks were obtained form the tissue bank of PLA General Hospital. Immunofluorescent (IF) staining was performed in KIRC tissues. Briefly, The slices were incubated with indicated primary anti-RARRES1(#MA5-26247, Invitrogen), anti-ICAM-1 (#10831-1-AP, Proteintech) and anti-CD86(#ab239075, Abcam) at 4°C overnight and then incubated with Cy3-conjugated goat anti-rabbit IgG(#A0516, Beytime Biotechnology) secondary antibody or FITC-conjugated goat anti-mouse IgG(#A0568, Beytime Biotechnology) secondary antibody. The nucleus was stained with DAPI and signals were observed using confocal fluorescence microscopy (Olympus). The evaluation was done *via* Image J software.

### Cell migration assay

Transwell migration chambers (8.0 μm pore diamete, Corning Incorporated) were used to evaluated the migratory capacity of M1 THP-1 cells. The M1 THP-1 cells and Caki-1 cells were cultured in DMEM medium (HyClone™) supplemented with 10% FBS(Gibco™). M1 THP-1 cells were seed in the upper chambers at 2×10^5^ cells/well, and Caki-1 cells were grown in the lower chambers at 15×10^5^ cells/well. After incubated for 24h at 37°C in an incubator, the upper chamber was removed, and the cells were fixed with 4% paraformaldehyde. We used a cotton swab to remove cells on the upper surface of the filter membrane. 0.1% crystal violet (#G106, Solarbio) was used to stain the cells migrated to the lower surface. Image J software was used to record migrated cells. Cells were counted and averaged in 5 random fields.

### Cell viability assay

Caki-1 cells were cultured in a 96-well plate (2000 cells/well) for 12 h and then cocultured with or without M1 THP-1 cells in the Transwell system for anther 24 h. The culture medium added 10% CCK-8 solution(#CK04, Dojindo) and incubated in 37°C temperature. Cell viability was quantified by evaluating the OD450 with a microplate reader.

### Western blot analysis

Total protein extracted from cells were used RIPA buffer. Proteins were separated by 10% SDS-PAGE and then transferred to a PVDF membrane. After blocking by 5% skim milk for one hour, the membrane was incubated with an anti-RARRES1(#MA5-26247, Invitrogen), anti-CD86(#ab239075, Abcam), anti-CD68(#ab213363, Abcam) and anti-cleaved caspase-3 primary antibody (#ab2302, Abcam). HRG-tagged anti-rabbit IgG (#ab205718, Abcam) was used as the secondary antibody. Immunoreactive bands were detected with the Western blot reagent ECL and the gray values were analyzed with ImageJ software.

### Statistical analysis

UALCAN and GEPIA gene expression data were analyzed using Student’s t test. Spearman’s correlation analysis was used for evaluation of correlation analysis. Database-derived tools were applied to all statistical tests. Statistical analysis was performed with GraphPad Prism 8.0 (GraphPad Software Inc., San Diego, CA, USA). The results are expressed as the mean ± standard deviation of at least 3 independent experiments. One-way ANOVA was performed with the Bonferroni method for multiple comparisons. The t test was used for comparisons between two groups.

## Results

### RARRES1 was related to the prognosis of KIRC

**Figure 1:**
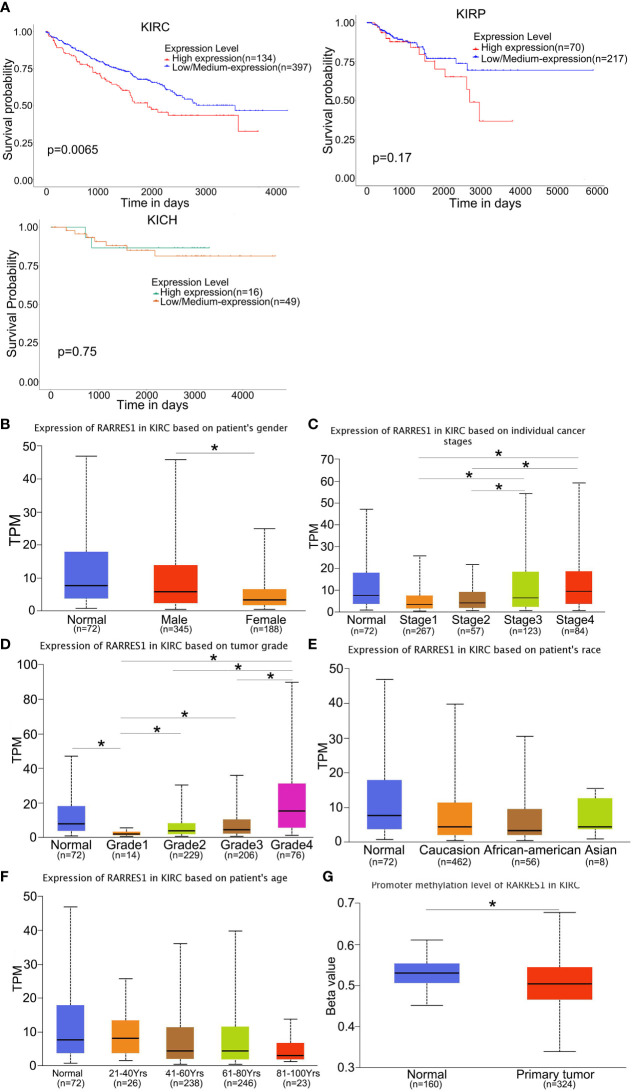
RARRES1 expression in KIRC. **(A)** The effect of the RARRES1 expression level on KIRC, KIRP and KICH patient survival was assessed by Kaplan–Meier survival analysis. **(B–G)** The differential expression of RARRES1 in KIRC patients. RARRES1 expression in different subgroups. **(B)** The expression of RARRES1 in male patients was significantly different from that in female patients (p=0.00144). **(C)** The RARRES1 expression level in the stage 1 subgroup was significantly different from those in the stage 3 (p=0.037) and stage 4 (p=0.0017) subgroups. The RARRES1 expression level in the stage 2 subgroup was significantly different from those in the stage 3 (p=0.039) and stage 4 (p=0.0056) subgroups. **(D)** RARRES1 expression in different tumor grades. **(E)** Expression of RARRES1 in KIRC based on race. RARRES1 expression in normal controls was not significantly different from that in Caucasian, African-American and Asian patients (p>0.05). **(F)** Expression of RARRES1 in KIRC based on age. There was no significant difference in RARRES1 expression among the groups (p>0.05). **(G)** The promoter methylation level in the RARRES1 gene was significantly decreased compared with that in normal controls (p < 0.0001) (TPM, transcripts per million). *p<0.05.

We analyzed survival rates by the KM curves, which indicated that RARRES1 expression was closely related to survival time in KIRC patients and that lower RARRES1 expression implied a longer survival time for patients. However, there was no correlation between RARRES1 expression and survival time in KIRP or KICH patients (P>0.05) (**Figure 1A**). To analyze RARRES1 expression in subgroups, we divided KIRC patients stratified by RARRES1 expression into subgroups based on age, race, sex, pathological grade, and tumor stage. The expression level of RARRES1 was significantly higher in male compared to female patients (p<0.01; **Figure 1B**). The expression level of RARRES1 in the subgroup of individuals with stage 1 tumors was significantly lower than that in the stage 3/4 subgroups, and the RARRES1 expression level in the stage 2 subgroup was also significantly lower than that in the stage 3 and stage 4 subgroups (p<0.05) (**Figure 1C**. The expression of RARRES1 was significantly lower in the tumor grade 1 subgroup than in the normal subgroup and the tumor grade 2/3/4 subgroup (P<0.001). In addition, the RARRES1 expression level was significantly lower in the tumor grade 2 and 3 subgroups than in the tumor grade 4 subgroup (**Figure 1D**). There was no significant difference in RARRES1 expression among the normal control, Caucasian, African-American and Asian populations (p>0.05). (**Figure 1E**). Patients were separated into four subgroups by age with a 20-year interval, and there was no significant difference among the age 21-40, 41-60, 61-80 and 81-100 subgroups (p>0.05) (**Figure 1F**). DNA methylation is an epigenetic regulation mechanism involved in gene transcription and tissue development. The methylation level of the RARRES1 gene was decreased in KIRC tissue samples (p<0.0001) (**Figure 1G**).

### Regulation of cell adhesion was the GO biological process most significantly enriched with genes related to RARRES1

**Figure 2:**
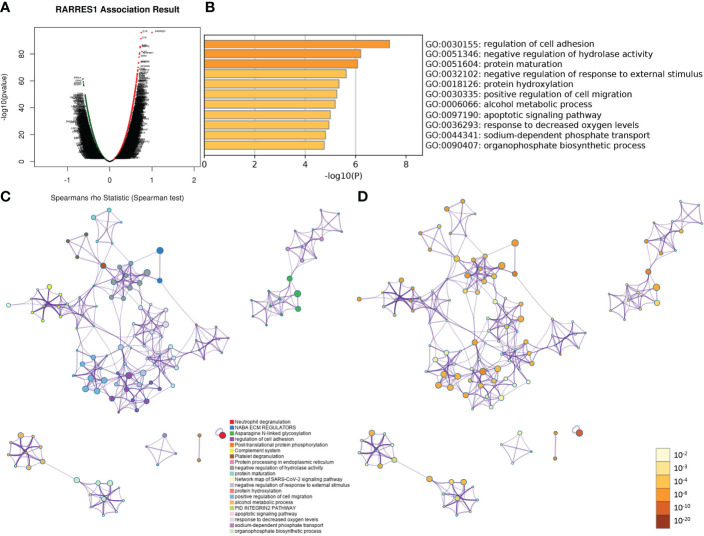
GO and PPI analyses of RARRES1-correlated genes. **(A)** The genes highly correlated with RARRES1 identified by Pearson correlation analysis in the KIRC cohort. **(B)** Main GO biological processes of all 284 genes correlated with RARRES1. **(C)** The PPI networks determined by functional cluster analysis. The different colors represent different functional clusters. **(D)** The PPI networks determined by functional cluster analysis. The different colors represent different P values. GO, Gene Ontology. PPI, protein–protein interaction.

As gene regulatory networks suggest genetic risk factors that have functional relationships, we studied the regulatory factors of RARRES1 in KIRC. **Figure 2A** shows the genes highly coexpressed with RARRES1; 164 genes were positively correlated with RARRES1(dark red dots), and 120 genes were negatively correlated with RARRES1(dark green dots). After that, we used the Metascape analysis tool to analyze RARRES1 and the 284 related genes and found that the main enriched GO biological processes were regulation of cell adhesion, protein maturation, negative regulation of hydrolase activity, negative regulation of response to external stimuli, protein hydroxylation, and positive regulation of cell migration (**Figure 2B**). A similar effect was seen in PPI networks identified by functional cluster analysis (**Figures 2C, D**). The GO biological process most significantly enriched with genes related to RARRES1 was regulation of cell adhesion. It is well known that cell adhesion is the first step in cancer metastasis and invasion. Twenty-six genes were included in the GO biological process regulation of cell adhesion: ADA, BCL2, ICAM1 and so on.

### Eight hub genes overlapped between the GO term regulation of cell adhesion and immune-related genes

**Figure 3:**
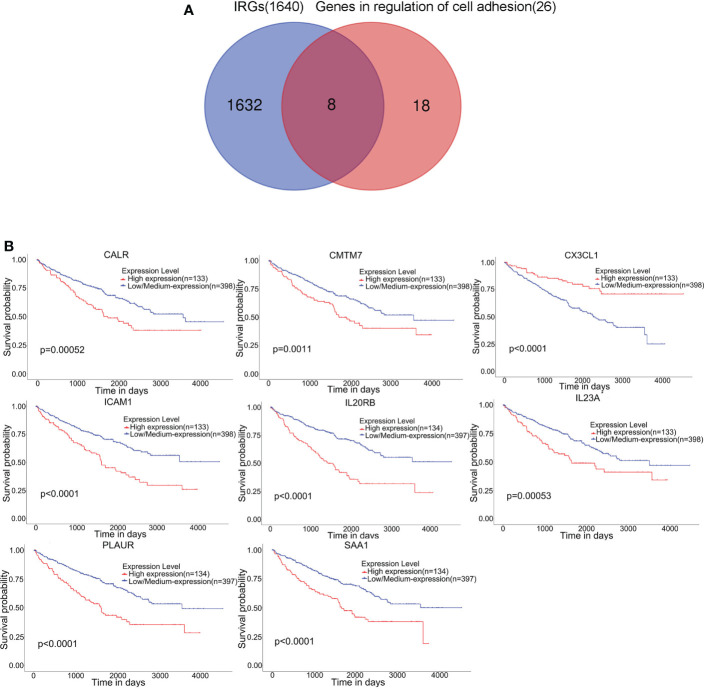
Eight hub genes were found in the regulation of cell adhesion GO term and among immune-related genes (IRGs). **(A)** Identification of 8 common genes between the GO biological process regulation of cell adhesion and the set of immune-related genes (IRGs). The different colored regions represent different datasets. The overlapping region indicates overlapping genes. **(B)** Kaplan–Meier survival analysis based on the expression of these 8 hub genes in KIRC was performed with the UALCAN database.

A total of 1640 immune-related genes (IRGs) were downloaded from the ImmPort database. By overlapping these IRGs with the 26 genes involved in the regulation of cell adhesion, which was the GO biological process most significantly enriched with genes related to RARRES1, we finally identified 8 common genes: CALR, ICAM1, CMTM7, CX3CL1, SAA1, PLAUR, IL23A, and IL20RB (**Figure 3A**). After that, the UALCAN database was used to analyze overall survival rates based on these 8 hub genes, and we found that high expression of these 8 hub genes are associated with poor prognosis(**Figure 3B**).

### ICAM1 had a relatively high correlation with RARRES1 among the 8 hub genes

**Figure 4:**
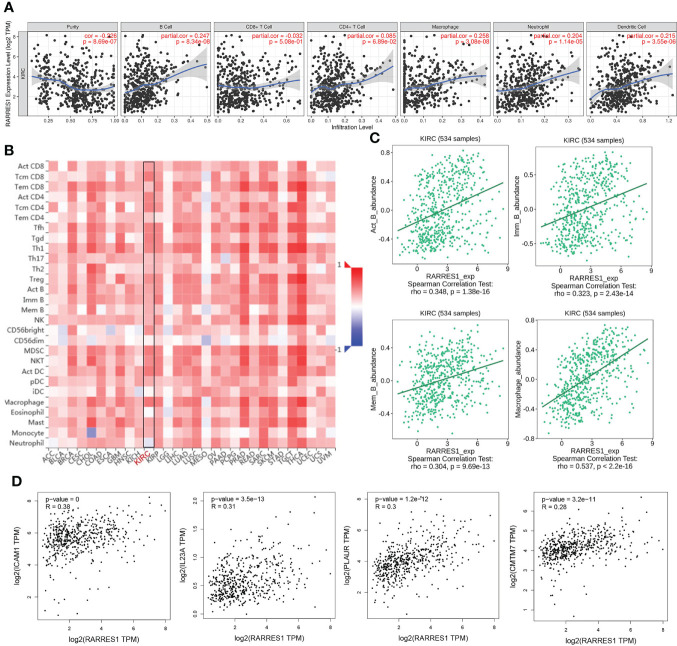
Analysis of associations between immune infiltration and the expression of RARRES1 and the 8 hub genes in KIRC. **(A)** Correlation of RARRES1 expression with the numbers of 6 tumor-infiltrating immune cells in KIRC using TIMER. **(B)** Relations between the expression of RARRES1 and the abundance of 28 types of TILs across heterogeneous human cancers. **(C)** RARRES1 expression was significantly correlated with the abundance of activated B cells (Act_B cells; rho=0.348, p<0.001), immature B cells (Imm_B cells; rho=0.323, p<0.001), memory B cells (Mem_B cells; rho=0.304, p<0.001), and macrophages (rho=0.537, p<0.001). **(D)** Correlations between the mRNA expression level of RARRES1 and those of CMTM7, PLAUR, IL23A, and ICAM1 in KIRC were determined using GEPIA.

According to TIMER, a web tool for analyzing immune cell infiltration in TCGA data, we discovered that RARRES1 expression had a negative correlation with tumor purity in KIRC and strong positive correlations with the infiltration of B cells and macrophages. The expression level of RARRES1 was positively correlated with the infiltration levels of B cells (r=0.247, P=8.34e-08), macrophages (r=0.258, P=3.08e-08), neutrophils (r=0.204, P=1.14e-05) and DCs (r=0.215, P=3.55e-06) in KIRC tissues (**Figure 4A**). In addition, we found that RARRES1 expression was significantly related to the abundance of 28 types of TILs in heterogeneous human cancers (**Figure 4B**). Regarding B cells and macrophages, RARRES1 expression was significantly related to the numbers of activated B cells Act_B cells; rho=0.348, p<0.001), immature B cells (Imm_B cells; rho=0.323, p<0.001), memory B cells (Mem_B cells; rho=0.304, p<0.001) and macrophages (rho=0.537, p<0.001) (**Figure 4C**). Among these cells, RARRES1 had the highest correlation with macrophages. We further analyzed the correlations between macrophage infiltration and the expression of the 8 hub genes related to RARRES1. As shown in **Table 1**, the expression levels of CMTM7, PLAUR, IL23A, and ICAM1 in KIRC tissues were significantly positively correlated with the infiltration of macrophages (p<0.05). We next analyzed the correlations between RARRES1 expression and CMTM7, PLAUR, IL23A, and ICAM1 expression with GEPIA. There was a relatively highest correlation (Spearman correlation coefficient=0.38) between RARRES1 and ICAM1 (CMTM7 = 0.28, PLAUR=0.3, and IL23A=0.31) (**Figure 4D**).

**Table 1:** Gene expression positively correlates with the infiltration level of macrophages in KIRC tissues.

variable	GENE	partial.cor	P value
Macrophage	ICAM1	0.201318588	1.76E-05
	CMTM7	0.328467814	9.91E-13
	PLAUR	0.271638741	5.11E-09
	IL23A	0.175304878	0.000192199
	CX3CL1	0.074709859	0.114313278
	CALR	0.063240745	0.181499461
	IL20RB	0.039214584	0.407661468
	SAA1	0.029919598	0.527615135

### ICAM1 was positively correlated with macrophage infiltration in KIRC

**Figure 5:**
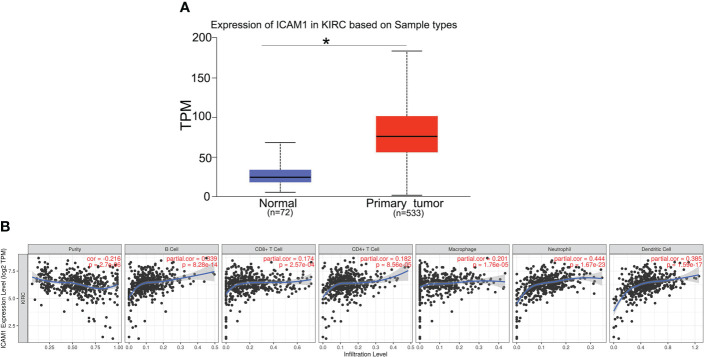
Expression of ICAM1 in KIRC patients. **(A)** ICAM1 was upregulated in primary tumors (p < 0.001). **(B)** Correlation analysis of ICAM1 expression and infiltration levels of immune cells in KIRC tissues using TIMER. ICAM1 expression in KIRC tissues was negatively correlated with tumor purity and positively correlated with the infiltration levels of B cells, CD8+ T cells, CD4+ T cells, macrophages, neutrophils, and dendritic cells. (KIRC, kidney renal clear cell carcinoma; TPM, transcripts per million.). *p<0.05.

Furthermore, we analyzed the ICAM1 expression in KIRC using the UALCAN database. Compared with normal controls group, ICAM1 expression was significantly increased in KIRC patients (**Figure 5A**). ICAM1 was highly correlated with patient survival, as shown by the KM curves (P<0.001) (**Figure 3B**). The ICAM1 expression was negatively correlated with tumor purity in KIRC and positively correlated with the infiltration levels of B cells (r=0.339, P=8.28e-14), CD8+ T cells (r=0.174, P=2.57e-04), CD4+ T cells (r=0.182, P=8.56e-05), macrophages (r=0.201, P=1.76e-05), neutrophils (r=0.444, P=1.67e-23), and DCs (r=0.385, P=1.59e-17) in KIRC tissues (**Figure 5B**). These results also indicated that ICAM1 was closely related to macrophage infiltration in KIRC.

### ICAM1 expression and interaction with Mac-1 were induced by RARRES1 overexpression

**Figure 6:**
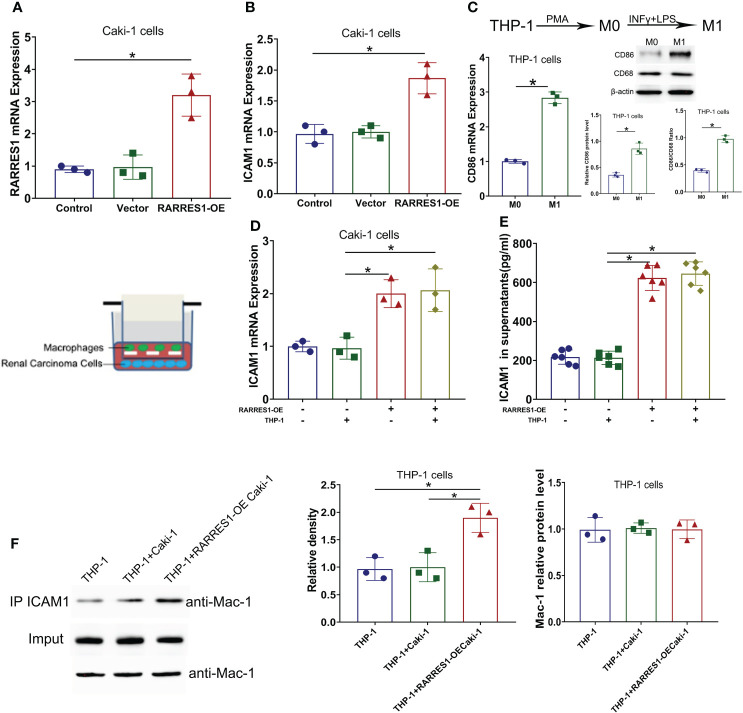
ICAM1 expression and interaction with Mac-1 were induced by RARRES1 overexpression. **(A, B)** Relative RARRES1 **(A)** and ICAM1 **(B)** mRNA expression in renal carcinoma cells, as measured by qPCR after transfection. RARRES1 and ICAM1 were significantly upregulated in the RARRES1-OE group; *p < 0.05 compared to the vector group; n = 3 in each group. **(C)** CD86 mRNA and protein expression in THP-1 cells after stimulation with IFN-γ. *p < 0.05 compared to the THP-1 only group. **(D)** ICAM1 mRNA expression in RCC cells cocultured with macrophages. Coculture of RARRES1-overexpressing RCC cells with macrophages induced evident upregulation of ICAM1 in RCC cells. *p < 0.05 **(E)** The level of ICAM1 in cell supernatants was measured by ELISA and was significantly increased after RARRES1 overexpression in RCC cells. *p < 0.05 **(F)** The Co-IP assay showed that overexpression of RARRES1 in RCC cells significantly promoted the interaction of ICAM1 and Mac-1. *p < 0.05.

GEPIA analysis indicated a positive correlation between RARRES1 and ICAM1 expression. To investigate whether RARRES1 overexpression can induce the expression of ICAM1 in RCC (Caki-1) cells, we generated RARRES1 overexpression lentivirus to infect RCC cells. Compared with control cells, RARRES1-overexpressing RCC cells showed significantly upregulated expression of RARRES1 and ICAM1 (**Figures 6A, B**). It is commonly considered that M1 macrophages contribute to the promotion of inflammation and tumor suppression. To investigate the interaction between M1 macrophages and renal carcinoma cells, THP-1 cells were induced to differentiate with IFN-γ+LPS, which act as activators of the M1 macrophage phenotype. IFN-γ+LPS stimulation increased the expression of CD86 at the mRNA and protein levels, suggesting that the polarization of THP-1 cells toward M1-like macrophages was successfully induced (**Figure 6C**). Given that Mac-1 (CD11b/CD18), mainly expressed in macrophages, is the receptor for ICAM1, to study whether RARRES1-overexpressing RCC cells can enhance the binding of ICAM1 to Mac-1 in macrophages, we cocultured RARRES1-overexpressing RCC cells and M1 macrophages in a Transwell system. The mRNA expression level of ICAM1 and the concentration of ICAM1 in cell supernatants were significantly increased when RARRES1 was overexpressed in RCC cells (**Figures 6D, E**). Next, we performed a Co-IP assay to examine the binding of ICAM1 and Mac-1 in macrophages.We found that the expression of Mac-1 in THP-1 cells did not change when THP-1 cells co-cultured with or without Caki-1 cells and RARRES1-OE Caki-1 cells. But the binding of ICAM1 with Mac-1 on THP-1 cells was increased with after RARRES1 was overexpressed in Caki-1 cells. These results demonstrated that overexpression of RARRES1 in RCC cells promoted the binding of ICAM1 and Mac-1 (**Figure 6F**).

### Validated the expression of RARRES1, ICAM1 and CD86 in KIRC tumor tissue

**Figure 7:**
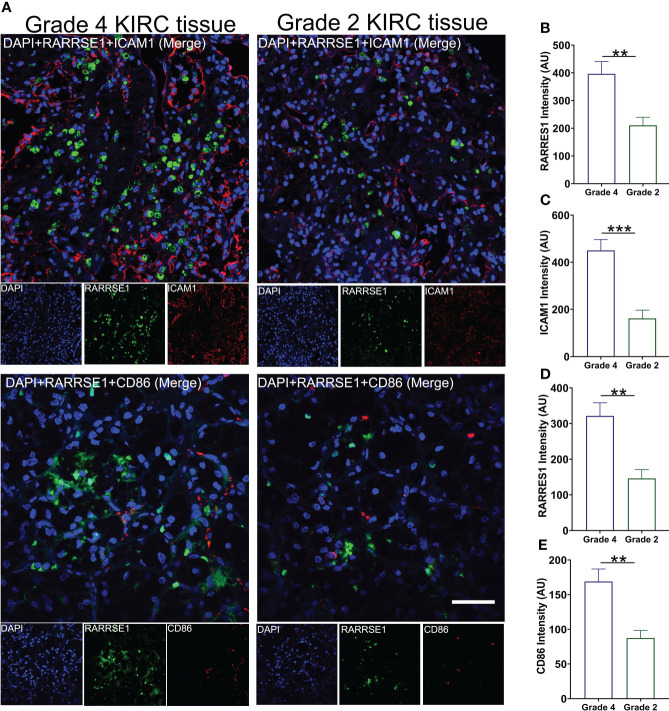
Validate the expression of RARRES1, ICAM1 and CD86 in KIRC tumor tissue. **(A)** The immunofluorescence staining of RARRES1, ICAM1 and CD86 in Grade 4 and Grade 2 KIRC samples (Scale bar, 50 μm). Fluorescence intensity of RARRES1 **(B)** combine with ICAM1 **(C)** in Grade 4 and 2 KIRC tissue groups. Fluorescence intensity of RARRES1 **(D)** combine with CD86 **(E)** in Grade 4 and 2 KIRC tissue groups. n = 3 in each group; **p < 0.01,***p < 0.001.

We also used KIRC samples to verify the correlation between RARRES1, ICAM1 and CD86. we performed immunofluorescence assay with Grade 4 KIRC tissues and Grade 2 KIRC tissues to study the expression difference. We found that the fluorescent signals of RARRES1 were stronger in Grade 4 KIRC tissues. Higher RARRES1 were accompanied by higher ICAM1 staining and higher CD86 staining. On the contrary, lower RARRES1 were accompanied by lower ICAM1 staining and lower CD86 staining. This is consistent with our above results which indicated that higher expression of RARRES1 may lead to more M1 macrophages infiltration (**Figure 7**).

### Knockdown of ICAM1 or Mac-1 decreased the migration potential of macrophages induced by RARRES1 overexpression

**Figure 8:**
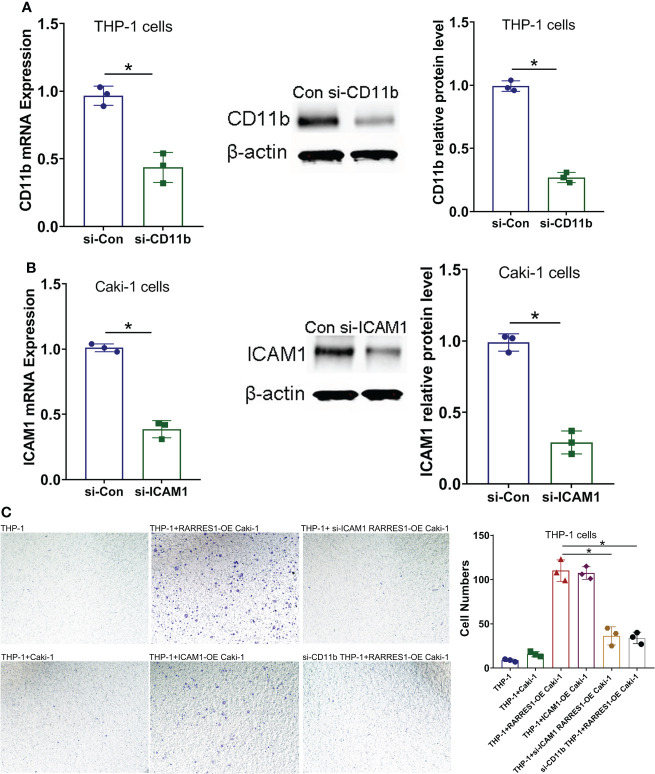
Blockade of Mac-1 or ICAM1 decreases the migration potential of macrophages. **(A, B)** The effects of siRNA transfection on Mac-1 (CD11b) and ICAM1 silencing were confirmed by qPCR and Western blot analysis. **(C)** Blockade of Mac-1 (CD11b) in macrophages or ICAM1 in RCC cells decreased RARRES1-OE-induced macrophage migration. The migration potential of macrophages (THP-1 cells) cocultured with renal carcinoma cells (Caki-1) was examined using a Transwell assay (scale bars: 100 μm). *p < 0.05.

To examine whether the Mac-1 and ICAM1 interaction affected the migration potential of macrophages after RARRES1 was overexpressed in RCC cells, we transfected siRNA-CD11b into macrophages and transfected siRNA-ICAM1 into RCC cells. As shown in **Figures 8A, B**, the mRNA and protein expression levels of CD11b in macrophages and ICAM1 in RCC cells were decreased after siRNA transfection. We assessed the migration ability of macrophages by coculture with RCC cells. The migration assay showed that the number of migrated macrophages was significantly increased after coculture with RARRES1-overexpressing RCC cells for 24 h, which demonstrated that RARRES1 overexpression (OE) in RCC cells promoted the migration of macrophages. Furthermore, after coculture with ICAM1-overexpressing (OE) RCC cells for 24 h, the number of migrated macrophages was significantly increased. Then, we evaluated the migration of macrophages after Mac-1 or ICAM1 was inhibited. The number of migrated macrophages was greatly reduced as CD11b was silenced in macrophages or ICAM1 was silenced in RCC cells (p<0.05) (**Figure 8C**). Taken together, these findings indicated that blockade of Mac-1 in macrophages or ICAM1 in RCC cells decreased macrophage migration induced by RARRES1-OE.

### M1-Like macrophages reduced the viability of renal carcinoma cells and induce apoptosis

**Figure 9:**
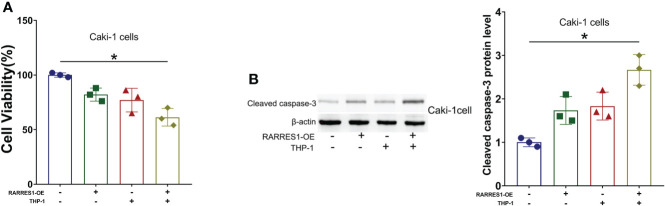
RARRES1-overexpressing RCC cell-induced M1 macrophages reduce the viability, induce the apoptosis and suppress the proliferation of renal carcinoma cells. **(A)** Cell viability was evaluated by a CCK-8 assay. **(B)** The level of an apoptosis-related protein (cleaved caspase-3) was measured by Western blot analysis. *p < 0.05.

We cocultured M1 macrophages with RCC cells to determine whether overexpression of RARRES1 can induce M1 macrophage polarization and mediate resistance to tumors. We aimed to explicit the effect of M1 type macrophages on the viability of cancer cells. The CCK-8 assay results showed that M1 macrophages significantly decreased the viability of renal carcinoma cells after being cocultured with RARRES1-overexpressing RCC cells (**Figure 9A**). In addition, the apoptosis rate of RCC cells was increased after M1 macrophages were cocultured with RARRES1-overexpressing RCC cells. In the RARRES1-overexpressing RCC cell group, the protein expression level of cleaved caspase-3 was significantly increased after co-culture with M1 macrophages compared with that in the group of RCC cells without RARRES1 overexpression (**Figure 9B**).

## Discussion

KIRC is the most common renal tumor. Genetic alterations or epigenetic regulation are involved in the progression and tumor environment of KIRC (19). RARRES1 acted as an invasion suppressor in prostate cancer and triple-negative breast cancer (20, 21). In our research, we assessed the prognostic value of RARRES1 in KIRC and observed that RARRES1 expression was negatively correlated with survival time. In our vitro experiments, we showed that RARRES1 exerts anti-tumor effect on tumor cells through macrophages. This seems inconsistent with KM curve of RARRES1 expression in KIRC. The reason maybe that tumors are abnormal organs composed of multiple cell types and extracellular matrix rather than simply clones of cancer cells (22). In our research, high expression of RARRES1 may indicate high degree of tumor malignancy and RARRES1 is recruiting more macrophages to suppress tumor. But the tumor microenvironment is complex, the tumor suppressor effects of RARRES1 may fail to counteract malignant tumor. These results provide insight into the influence of RARRES1 expression on the disease progression of KIRC through macrophage activation. We sought to determine the mechanism by which RARRES1 exerts a tumor-suppressive effect in KIRC.

Functional enrichment analysis demonstrated that the GO biological process most significantly enriched with genes related to RARRES1 was regulation of cell adhesion. Inflammation as a basic physiological process is a hallmark of various tumors (23). Cancer-associated inflammation involves reciprocal autocrine and paracrine communication among malignant and nonmalignant cells through chemokines, cytokines and prostaglandins. Inflammatory tumor environment combines with genetic alterations that ultimately lead to tumor progression and metastasis (24). Cell adhesion mediates cell–cell crosstalk and exerts an important effect on the inflammatory response. Intercellular adhesion molecule 1 (ICAM1) is regarded as a cell surface glycoprotein which is in the immunoglobulin superfamily that mediates adherence-dependent interactions between cells (25). Different expression levels of ICAM1 have been found in various malignant tumors. In this study, we found that ICAM1 is significantly upregulated in KIRC. ICAM1 can also regulate immune cell-mediated tumor cytotoxicity, thereby improving the prognosis of patients with certain tumors, such as melanoma and oral squamous cell carcinoma (26). Several articles have suggested that patients with ICAM1-positive tumor cells have better clinical outcomes in breast, colorectal, and gastric cancers (27). We analyzed the correlation between RARRES1 and ICAM1 expression. GEPIA analysis indicated that there was a relatively highest correlation between RARRES1 and ICAM1. Thus, we hypothesized that RARRES1 overexpression can upregulate the expression of ICAM1 and inhibit tumorigenesis. In addition, ICAM1 is an adhesion receptor that is best known for regulating immune cell recruitment (28).

Our study reveals that overexpression of RARRES1 can promote ICAM1 expression in RCC cells. In addition, we found that ICAM1 expression was negatively correlated with tumor purity in KIRC and positively correlated with the infiltration level of macrophages in KIRC. Macrophage antigen-1 (Mac-1, CD11b/CD18, CR3), a β2 integrin expressed on macrophages, is a receptor for ICAM1 (29). KIRC is a highly immunogenic tumor type whose tumor cells generate an immunosuppressive environment through multiple immunosuppressive mechanisms. KIRC tumors are surrounded by many inflammatory cells, such as T cells, NK cells, and macrophages (30). This observation could help to design new clinical trials for patients undergoing immunotherapy. Depending on the patterns of M1 and M2 polarization, macrophages probably play either a tumor-promoting role or a antitumor role (31). In RCC cells, M1 macrophage markers are expressed alongside M2 markers in tumor-Associated macrophages(TAM), suggesting that some TAMs can exhibit hybrid phenotypes in some cancers (32). M1-like macrophages are essential tumor suppressor cells that initially act in the inhibition of tumor cell growth in the tumor microenvironment (33).

For the application of novel immunotherapy, macrophage-based therapies could augment macrophage functionalities with antitumor activity (34). We found that both ICAM1 expression in RCC cells and Mac-1 expression in M1 macrophages were upregulated after RARRES1-overexpressing RCC cells were cocultured with macrophages. In addition, RARRES1 overexpression in renal carcinoma cells enhanced the migration potential of M1 macrophages. Blocking Mac-1 in M1 macrophages or ICAM1 in RCC decreased RARRES1-OE-induced M1 macrophage migration. We showed that M1 macrophages more significantly decreased the viability of RARRES1-overexpressing renal carcinoma cells and increased the apoptosis rate of renal carcinoma cells. Collectively, these results indicate that M1 type macrophages perform antitumor functions by decreasing the viability of renal carcinoma cells and inducing their apoptosis. Our results indicated interaction of RARRES1 with ICAM1 modulating macrophages may be a new target for immunotherapy of kidney renal clear cell carcinoma.

In summary, RARRES1 expression is strongly related to cancer progression, survival rate and immune invasion in KIRC patients. According to the bioinformatics analysis and preliminary validation experiments, we suggest that the antitumor effect of RARRES1 is achieved by promoting the expression of ICAM1 and inducing the activation of M1 macrophages. This study offers promising insights for subsequent research to elucidate the molecular pathogenesis of KIRC.

## Data availability statement

The datasets presented in this study can be found in online repositories. The names of the repository/repositories and accession numbers can be found within the article/Supplementary Materials.

## Ethics statement

The studies involving human participants were reviewed and approved by PLA General Hospital ethics committee. The patients/participants provided their written informed consent to participate in this study. The animal study was reviewed and approved by the Ethics Committee for the Use of Animals of PLA General Hospital.

## Author contributions

QH, GC, and XC conceived and designed the experiments. XG, KC, CL, ZF and HW performed the experiments. XW and LM analyzed the data. XG and KC wrote the manuscript. QH revised the manuscript. QH provided funding. All authors contributed to the article and approved the submitted version.

## Funding

Project supported by: Fostering Fund of Chinese PLA General Hospital for National Distinguished Young Scholar Science Fund (2019-JQPY-002), National Natural Science Foundation of China (NO.82204744, 82270758 and 82070741) and National Key Research and Development Project (2018YFE0126600).

## Conflict of interest

The authors declare that the research was conducted in the absence of any commercial or financial relationships that could be construed as a potential conflict of interest.

## Publisher’s note

All claims expressed in this article are solely those of the authors and do not necessarily represent those of their affiliated organizations, or those of the publisher, the editors and the reviewers. Any product that may be evaluated in this article, or claim that may be made by its manufacturer, is not guaranteed or endorsed by the publisher.
